# Inferring predominant pathways in cellular models of breast cancer using limited sample proteomic profiling

**DOI:** 10.1186/1471-2407-10-291

**Published:** 2010-06-15

**Authors:** Yogesh M Kulkarni, Vivian Suarez, David J Klinke

**Affiliations:** 1 Department of Chemical Engineering, West Virginia University College of Engineering and Mineral Resources, West Virginia University, Morgantown, WV, 26506, USA; 2 Department of Microbiology, Immunology and Cell Biology, West Virginia University School of Medicine, West Virginia University, Morgantown, WV, 26506, USA

## Abstract

**Background:**

Molecularly targeted drugs inhibit aberrant signaling within oncogenic pathways. Identifying the predominant pathways at work within a tumor is a key step towards tailoring therapies to the patient. Clinical samples pose significant challenges for proteomic profiling, an attractive approach for identifying predominant pathways. The objective of this study was to determine if information obtained from a limited sample (i.e., a single gel replicate) can provide insight into the predominant pathways in two well-characterized breast cancer models.

**Methods:**

A comparative proteomic analysis of total cell lysates was obtained from two cellular models of breast cancer, BT474 (HER2+/ER+) and SKBR3 (HER2+/ER-), using two-dimensional electrophoresis and MALDI-TOF mass spectrometry. Protein interaction networks and canonical pathways were extracted from the Ingenuity Pathway Knowledgebase (IPK) based on association with the observed pattern of differentially expressed proteins.

**Results:**

Of the 304 spots that were picked, 167 protein spots were identified. A threshold of 1.5-fold was used to select 62 proteins used in the analysis. IPK analysis suggested that metabolic pathways were highly associated with protein expression in SKBR3 cells while cell motility pathways were highly associated with BT474 cells. Inferred protein networks were confirmed by observing an up-regulation of IGF-1R and profilin in BT474 and up-regulation of Ras and enolase in SKBR3 using western blot.

**Conclusion:**

When interpreted in the context of prior information, our results suggest that the overall patterns of differential protein expression obtained from limited samples can still aid in clinical decision making by providing an estimate of the predominant pathways that underpin cellular phenotype.

## Background

Breast cancers are clinically heterogeneous [1]. Particular molecules have been identified that are associated with clinical prognosis. For instance, 20% to 25% of breast cancers are associated with the overexpression of HER2, and its presence is associated with poor prognosis [2,3]. In addition, increased ER/PR expression has been identified in 70% of breast cancer patients. These biomarkers have motivated a shift away from "one size fits all" approach of treating breast cancer to developing therapies that target specific molecules. In particular, tamoxifen, a selective ER modulator (SERM), improves survival for patients with ER/PR positive tumors [4,5]. Trastuzumab, a human monoclonal antibody was developed to bind the HER2 receptor and block its activity [6]. However, *de novo *or acquired resistance to tamoxifen [7-9] and trastuzumab [10] has been an emerging problem.

Molecularly targeted drugs, like trastuzumab and tamoxifen, are designed to block aberrant signaling within oncogenic pathways. Cell signaling pathways direct the flow of information (i.e. flux) from an extracellular stimulus to the corresponding cellular response (e.g., cellular proliferation, contact inhibition, or cellular death). By analogy with metabolic control analysis, control of flow of information is distributed among all the steps in a network [11-13]. This implies that an increase in one protein does not necessarily correspond to an increased pathway flux. Conversely, the decrease in expression of one protein via therapeutic modification does not necessarily lead to a decrease in pathway flux. Conceptually, this leads to the hypothesis that combining gene expression measurements over group of genes that fall within common pathways will be more effective means of marker identification. In fact, recently it was shown that breast cancer genes that do not exhibit a change in their expression profile still play a central role interconnecting deregulated genes in a protein network [14]. The observation that onset and progression of many diseases arises from the interactions of a number of interconnected genes has shifted the drug discovery perspective from a molecule-centric to a network/pathway-centric approach [15]. Proteomics provides an attractive platform for interrogating pathway flux as measuring actual protein levels instead of measuring proxy mRNA levels maybe more informative in spite of added experimental complexity [16].

One of the most commonly used techniques for proteomic profiling is 2DE based protein separation in combination with mass spectrometry based identification. Using this approach, in addition to analyzing proteins in the blood, tumor tissues are being examined to yield insights about molecular pathways that are altered in cancer progression. While 2-DE based high-throughput proteomic data reveal proteins that are differentially regulated, different sources of biological and analytical variations affect the statistical importance of these results [17]. These can be addressed using an experimental design that incorporates several technical and biological replicates to account for variations at two levels, within gels and within samples, respectively [18-20]. This puts a demand on the sample amount and composition as proteomic analysis of breast cancer biopsies is complicated due to heterogeneity of cellular phenotypes contained in the sample [21]. While laser capture microdissection (LCM) can provide a relatively homogeneous sample by concentrating on the cell type of interest, generating enough sample for a conventional proteomic study is laborious with a minimum of 100,000 cells and a dissection time in tens of hours required for one 2D-PAGE [22]. Given the desire to aid in clinical decision-making, the ability to obtain sufficient clinical sample presents a significant challenge. In the case of sample that is insufficient to carry out a proteomic study with multiple replicates, like in the case of an early stage breast tumor, does the information obtained from a single gel replicate still provide insight into the predominant cell signaling pathway at work in a cell?

Thus the objective of this study was to identify predominant pathways and protein interaction networks in two breast cancer phenotypes using prior information. Given the conservation of genetic information and marker expression between tumors and their corresponding cell lines [23-25], we have used well characterized model systems in our study. The central hubs of protein interaction networks obtained using prior information were validated to establish confidence in the protein expression patterns.

## Methods

### Cell culture and reagents

The human breast cancer cell lines (BT-474 and SK-BR-3) were kindly provided by Dr. Jia Luo (Health Sciences Center; West Virginia University, WV). Cells were grown in 75-cm^2 ^plastic tissue culture flasks (Costar Corning; Corning, NY) in a humidified incubator at 37°C and 5% (v/v) CO_2_. The BT-474 cells were routinely maintained in Rosewell Park Memorial Institute (RPMI) 1640 medium (Mediatech, Inc., Herndon, VA) supplemented with 10% (v/v) heat inactivated fetal bovine serum (FBS) (Hyclone, Inc., Logan, UT), 0.3% (w/v) L-glutamine, 1% (v/v) penicillin/streptomycin (BioWhittaker, Walkersville, MD) and 10 ng/mL insulin (Sigma, St Louis, MO). SK-BR-3 cells were maintained in Improved Modified Eagle Medium (IMEM) Zn^2+ ^option (Invitrogen) containing 4 mM L-glutamine, 2 ml/L L-proline, 50 μg/mL gentamicin sulfate supplemented with 10% FBS (Hyclone) and 1% penicillin/streptomycin (BioWhittaker). Cells were passaged at 1:5 dilution with fresh medium every 5 days.

### Preparation of cell lines for 2-DE

Cells were grown to approximately 80% confluence. Growth medium was removed from dishes and cells were washed twice with 10 mL Phosphate Buffered Saline (PBS) to remove dead cells as many extracellular proteins as possible. Cells were made non-adherent by incubating the flasks at 37°C for 10 min in the presence of trypsin (BioWhittaker). Trypsin was neutralized by the addition of FBS. Cells were then washed twice with PBS and harvested at 1,200 rpm at 4°C for 10 min. Sufficient precaution was taken to get rid of PBS to eliminate salts that could possibly interfere with the 2DE. Cells were incubated in lysis buffer (7M Urea, 2M thiourea, 2% (w/v) CHAPS) for 30 min on ice and sonicated five times in an ultrasonic water bath, where each sonication was performed for 10 s followed by 10 s cooling interval on ice. Cell debris were pelleted by centrifugation at 14,000 rpm for 40 min at 4°C. The supernatant was aliquoted in fresh tubes and stored at -80°C. The protein concentration was determined using BCA protein assay kit (Pierce).

### 2-D Electrophoresis

For each cell line, 500 μg of cell lysate was mixed with rehydration buffer (7M urea, 2M thiourea, 2% CHAPS, 1% DTT, 2% IPG buffer, 0.002% bromophenol blue) and incubated for 1 h at room temperature prior to rehydration on Immobilized pH Gradient (IPG) strips pH 3-10 NL, 24 cm, (GE Healthcare, Uppsala, Sweden) for 12 h at 25°C. Isoelectric focusing was done using Ettan IPGphor apparatus (Amersham Biosciences) for a total of 90 kVh at 50 μA per strip at 20°C. Thereafter, IPG strips were equilibrated in 75 mM Tris-HCl pH 8.8, 6M urea, 30% (v/v) glycerol, 2% (w/v) SDS, 0.002% (w/v) bromophenol blue and 1% (w/v) DTT for 30 min. A second equilibration step was done for another 30 min by replacing the DTT with 2.5% iodoacetamide. Equilibrated strips were transferred onto 24 cm 12% uniform precast SDS-polyacrylamide gels (Jule, Inc., Milford, CT) poured between non-fluorescent glass plates. IPG strips were sealed with 0.5% (w/v) low melting point agarose in SDS running buffer containing bromophenol blue. Gels were run in Ettan DALTsix Larger Vertical System (Amersham Biosciences) at 30mA per gel at room temperature, until the dye front had run off the bottom of the gels.

Gels were fixed in 10% (v/v) methanol, 7% (v/v) acetic acid overnight, washed in 18 MΩ water, and stained overnight with SYPRORuby dye (Bio-Rad). Excess dye was removed by washing twice with 18 MΩ water in a dark room. Gels were imaged using the Typhoon 9400 scanner (Amersham Biosciences) at 200 μm resolution with a 488nm laser with 610nm band pass filter at normal sensitivity under fluorescence acquisition mode. Data were saved in .gel format using ImageQuant software (Amersham Biosciences). The 2-DE results are representative of three biological replicates.

### Image analysis

The images were analyzed using SameSpots software from Nonlinear Dynamics. Saturated and damaged areas of the gels were ignored in the analysis by selecting a region of interest (ROI). The images were warped using automatic and manual vectors to a reference image that was automatically selected based on the gel containing the most spots. Normalized spot volumes were generated from the optical densities for each individual spot to the ratio of the total spot volume in each gel. 304 differentially expressed protein spots were chosen for further analysis.

### In-gel digestion

The gel spots of interest were excised using an Ettan Spot Picker (Amersham Biosciences) fitted with a 1.5-mm spot picker head. Briefly, specified excised spots were reduced in DTT (10 mM, 60°C, 10 min) and alkylated with iodoacetamide (100 mM, room temperature, 45 min) in a dark room. The gel pieces were dehydrated in acetonitrile for 10 min. Then the gel pieces were vacuum dried and rehydrated with 10 μL of digestion buffer (10 ng/μL of trypsin (Promega; Madison, WI) in 25 mM NH_4_HCO_3_) and covered with 10 μL of NH_4_HCO_3_. The samples were incubated for 16 h at 37°C to allow for complete digestion. Peptides were extracted from gel plugs by sonication in 2.5 μL 5% formic acid.

### MALDI-TOF MS analysis

MALDI-TOF-MS system model Micromass MALDI-R (Waters^®^) was used to obtain the peptide mass fragment spectra as recommended by the manufacturer. Protein digest solutions were mixed at a 1:1 ratio with the MALDI matrix α-cyano-4-hydroxycinnamic acid (CHCA) (Sigma-Aldrich Fluka; St. Louis, MO). 1 μL of tryptic peptide sample was applied to the MALDI plate and allowed to dry. The MALDI-TOF MS was operated in the positive ion delayed extraction reflector mode for highest resolution and mass accuracy. Peptides were ionized/desorbed with a 337-nm laser and spectra were acquired at 15 kV accelerating potential with optimized parameters. The close external calibration method employing a mixture of standard peptides (Applied Biosystems) provided mass accuracy of 25-50 ppm. Internal calibration was performed with the monoisotopic peak of adrenocorticotropic hormone (ACTH) (18-39) peptide (*m/z*: 2465.1989). Mass spectral analysis for each sample was based on the average of 300 laser shots. Peptide masses were measured from *m/z*: 800 to 3,000. The peak lists containing the *m/z *ratio and corresponding intensity values were exported to Microsoft Excel for further processing.

### Protein identification using peptide mass fingerprinting (PMF)

Peptide mass fingerprints for each of the 304 proteins were entered in an Excel spreadsheet along side each other. To optimize the database searching, the list of peptide mass peaks from the spectrum of each sample was processed and background peaks that were observed in greater than 10% of the PMF's were eliminated to improve the efficiency of database searching [26]. MASCOT http://www.matrixscience.com, Aldente (ExPASy) and MS-Fit (Protein Prospector; University of California, San Francisco) were each used to query the UniProtKB/Swiss-Prot human database with the corresponding monoisotopic peptide mass fingerprints with the following settings: peptide mass tolerance of 50 ppm, one missed cleavage site, one fixed modification of carboxymethyl cysteine, one variable modification of methionine oxidation, and no restrictions on protein molecular mass or isoelectric point. The protein identities reported were ranked high in at least two of the three algorithms used.

### Ingenuity pathway Analysis

Differentially regulated proteins identified by 2DE and PMF were further analyzed using Ingenuity Pathway Analysis (IPA; Ingenuity Systems, Mountain View, CA; http://www.ingenuity.com). IPA was used to interpret the differentially expressed proteins in terms of an interaction network and predominant canonical pathways. The Ingenuity Pathways Knowledge Base (IKB) is a regularly updated curated database that consists of interactions between different proteins culled from scientific literature. IPA uses this database to construct protein interaction clusters that involve direct and indirect interactions, physical binding interactions, enzyme-substrate relationships, and cis-trans relationships in transcriptional control. The networks are displayed graphically as nodes (proteins) and edges (the biological relationship between the proteins).

A protein interaction network was generated as follows. A dataset containing the upregulated proteins, called the focus proteins, for a particular cell line was uploaded into the IPA. These focus proteins were overlaid onto a global molecular network developed from the information in the IKB. Networks of these focus proteins were then algorithmically generated by including as many focus proteins as possible and other non-focus proteins from the IKB that are needed to generate the network based on connectivity.

Canonical pathways are identified from the IPA library based on their significance to the dataset. The significance of the association between the dataset and the canonical pathway is measured in two ways: a) a ratio of the number of proteins in the dataset that map to the pathway divided by the total number of proteins that exist in the canonical pathway and b) a *p*-value that is obtained by comparing the number of genes/proteins of interest relative (i.e., focus genes) to the total number of genes/proteins in all functional/pathway annotations stored in the Ingenuity Pathways knowledge base (i.e. a right-tailed Fisher's exact test of a 2 × 2 contingency table with the Benjamini-Hochberg correction for multiple hypothesis testing). The 2 × 2 contingency table is shown in Table 1, where K is the number of genes/proteins of interest (i.e., focus genes) and N is the total number of genes/proteins in all pathway annotations. This test is a standardized choice in the IPA estimate of statistically significant findings. The null hypothesis tested was that the pathways associated with the upregulated proteins were likely to be observed by random chance alone. A low p-value suggests that the pathways associated with the upregulated proteins were not observed by random chance alone.

**Table 1:** 2 × 2 contingency table used for testing the significance of gene/protein enrichment in all IKB pathway annotations.

	Focus Genes	Non-focus Genes	Row Total
Genes associated with pathway	k	n - k	n
Genes not associated with pathway	K - k	(N - n) - (K - k)	N - n

Column Total	K	N - K	N

### Western blotting

For western blot analysis, 10-30 μg of total cell lysate was separated by SDS-PAGE using a 12% Tris polyacrylamide gel with a 4% stacking gel at 75 V for 4 h. Proteins were transferred onto Bio Trace PVDF membrane (PALL Life Sciences; Pensacola, FL) at 42 V for 1.5 h. Blots were washed in Tris Buffered Saline (TBS) for 5 min at room temperature, blocked for 1 h in TBS + 0.1% Tween 20 (TBS/T) plus 5% dry milk at room temperature and then washed three times in TBS/T. Blots were incubated overnight at 4°C with primary antibodies specific for IGF-1R (sc-9038), α-Enolase (sc-100812), GAPDH (sc-25778) (all from Santa Cruz Santa Cruz, CA), Ras (BD Biosciences, 610001) and Profilin (Millipore, AB3891) in TBS/T plus 5% dry milk. The next day, blots were washed three times in TBS/T, incubated for 1 h at room temperature with anti-biotin (Cell Signaling Technology, Inc., Danvers, MA, 7727) and either a goat anti-mouse IgG-horseradish peroxidase (HRP) (BD BioSciences, 554002) or a goat anti-rabbit IgG-HRP (Sigma-Aldrich, A0545). Finally, the blots were washed three times in TBS/T, developed using LumiGLO reagent (Cell Signaling Technology, Inc., Danvers, MA, 7003) and bands were visualized on KODAK Biomax light film (Fisher Scientific). Densitometric analysis was performed using ImageJ software (National Institute of Health) and protein levels were normalized to GAPDH protein levels for each sample. Given the uncertainty in estimating the level of expression in both cell lines, an empirical Bayesian approach was used to establish the level of confidence associated with differential expression between BT474 and SKBR-3 given the available data [27]. Levels of expression were log-transformed to minimize potential bias in estimating the expression ratio, R, as follows: log_10 _(X_BT474_) = R + log_10 _(X_SKBR3_). A Markov chain Monte Carlo algorithm was used to estimate the posterior distribution in the differential expression coefficient. An initial unbiased gaussian prior distribution was used to propose new steps in the Markov chain. The prior distribution was scaled to achieve an acceptance fraction of 0.4. The Gelman-Rubin potential scale reduction factor was used to estimate convergence of three independent Markov chains to the posterior distribution [28]. Posterior estimates of the expression ratio were obtained from the tails of the three independent chains following convergence.

## Results

### Identification of differentially expressed proteins

#### 2DE and Image Analysis

**Figure 1:**
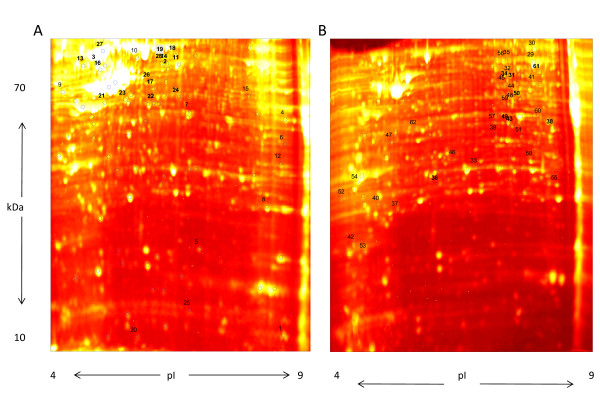
**Two-dimensional proteomic profile of breast cancer cell line A) BT474, B) SKBR3**. The first dimension represents a section of the gel spanning pH 4-9. The second dimension is a 12% PAGE spanning molecular weights 10-70 kDa. Gels were stained with SYPRO Ruby and imaged on a Typhoon 9400 scanner. The circles and squares in panel A and B, respectively, identify picked spots. Identified proteins are labeled with ID numbers, shown in Additional File 2, Table S1.

Three biological replicates for each cell line were obtained to ensure that the 2DE protocol provided consistent results. Qualitative criteria were used to determine the consistency among the three biological replicates (i.e., the images obtained from all three replicate were visually similar). A single replicate from each cell line was selected for further analysis. Figure 1 depicts a representative pattern of cellular proteome obtained after 2DE of total cellular extracts from BT474 (Figure 1A) and SKBR3 (Figure 1B) cell lines. Quantitative analysis was restricted to a region of interest that spanned an isoelectric focusing range of 4-9 and an approximate molecular weight range of 15 - 70 kDa. A total of 866 spots were identified in common between the gel images from each cell lines within this region of interest.

#### Peptide mass fingerprinting

Of the 866 spots identified to be common between both the gels using Progenesis SameSpots, spots that were not differentially expressed or detected in streaks were excluded from further analysis. 304 well resolved protein spots that were differentially expressed by at least 1.1-fold were excised from the gel, subjected to trypsin digestion and MALDI-TOF analysis. Using the resulting peptide mass fingerprints, 167 proteins were identified using Mascot as a primary database search algorithm, out of which 135 were unique. A subset of 62 identified proteins that were differentially regulated by at least 1.5-fold is shown in Additional file 1, Table II. More than 95% of the spots had sequence coverage exceeding 25%. In a majority of the cases, the identified proteins were the only candidate with significant score leading to their unambiguous identification. However, in some cases two different algorithms, MS-Fit and Aldente, were used to provide support for the protein identity from the PMF in order to minimize false positive identifications. Agreement between the apparent M_r _and pI observed from 2-DE gels and the theoretical values of the identified proteins provided additional support for positive identification. A small subset of the identified proteins exists in multiple forms, as they were associated with multiple protein spots. This may be due to either protein degradation by proteases and formation of protein fragments or post-translational modifications [29] such as phosphorylation and carbamylation which is common with proteins in urea buffer and/or different isotypes. A list of all the identified proteins with their molecular weight, isoelectric point, rank, score and sequence coverage using the three algorithms is provided (Additional file 2, Table S1).

#### Protein distribution according to function, subcellular localization and fold-change

**Figure 2:**
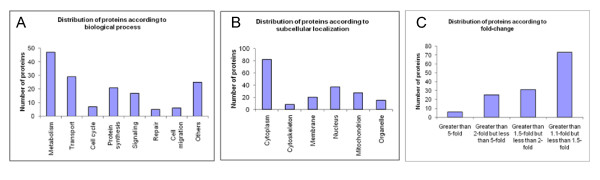
**Percent distribution of 135 uniquely identified proteins according to A) Biological process, B) Subcellular location, C) Fold-change according to GO annotation terms**.

Combining proteomics techniques with MALDI-TOF MS analysis enabled positive identification of 167 proteins, representing a broad range of functional and compartmental classes. The proteins were assigned a biological process and subcellular localization according to Gene Ontology (GO). Figure 2 displays the distribution of proteins across various biological processes (Figure 2A), subcellular compartments (Figure 2B) and fold-change in expression (Figure 2C) across the two cell lines. The largest fraction of identified proteins was enzymes that belonged to the metabolism group, necessary for the cell's ATP turnover. Other major groups of proteins involved initiation and elongation factors involved in protein synthesis, structural proteins like profilin and cofilin involved in the assembly and rearrangement of the cytoskeleton, and protection proteins that are involved in protecting the cell from oxidative stress (heat shock protein beta-1) and by degradation of misfolded proteins (UV excision repair protein RAD23 homolog B). Many proteins are multifunctional and shuttle between different cellular compartments in different cellular contexts. For example, Cofilin-1 is present in cytoplasm as well as nucleus and alpha-enolase is present in cytoplasm and cell membrane. Protein DJ-1 plays a role in transcription, acts as a chaperone and protects neurons against oxidative stress. Similarly, alpha-enolase is a multifunctional enzyme that plays a key role in glycolysis and in various processes such as growth control and hypoxia tolerance. The differentially expressed proteins represent a diverse sampling cross-section of different biological processes and subcellular compartments. Proteins deregulated by 1.5-fold shown in Additional file 1, Table II formed the dataset for pathway analysis and network generation.

### Pathway analysis and network generation using IPA

**Figure 3:**
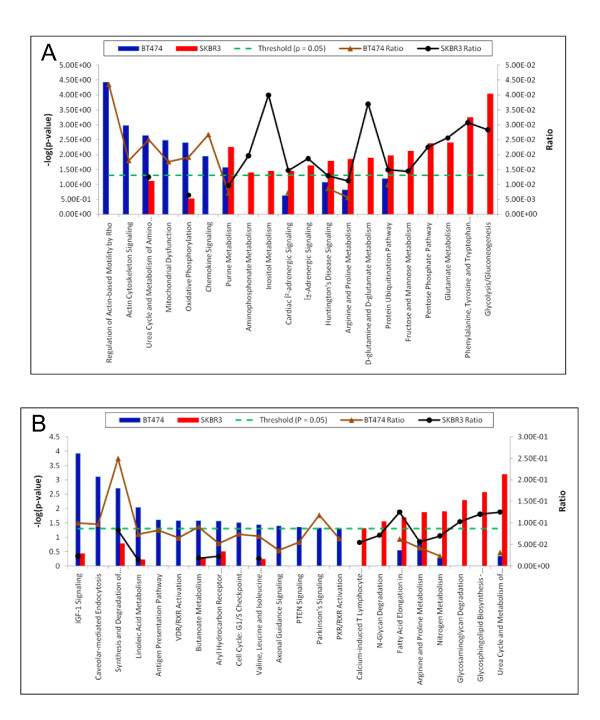
**Significant canonical pathways (*p*-value < 0.05) for BT474 (blue) and SKBR3 (red) cell lines using 1.5-fold A) deregulated proteins and B) deregulated genes**. The negative of the log_10_(p-value) and ratio are plotted on the primary and secondary Y-axis respectively.

Pathway analysis was performed to relate the differentially expressed proteins to canonical biological pathways. Canonical pathway analysis identified the pathways from the IPA library of canonical pathways that were most significant to the proteins expressed differentially. The prior information used in this analysis contains list of canonical pathways and associated proteins. A Benjamini-Hochberg corrected Fischer's exact test was used to calculate the p-values associated with a canonical pathway [30]. Additionally, a ratio determining the number of focus molecules to overall molecules in each pathway was also used to associate canonical pathways to the dataset. The results from this pathway analysis are summarized in Figure 3A. The most significant canonical pathway in BT474 cell line was Regulation of Actin-based Motility by Rho when ranked by significance (*p*-value < 3.7 × 10^-5^) as well as by ratio (4.35E-02). The most significant canonical pathway in SKBR3 cell line when ranked by significance (*p*-value < 9.1 × 10^-5^) was Glycolysis/Gluconeogenesis and when ranked by ratio (4.00E-02) was Inositol metabolism (*p*-value < 3.5 × 10^-2^). In general, deregulated proteins in BT474 and SKBR3 were found to be predominantly associated with cell motility pathways and metabolic pathways, respectively.

**Figure 4:**
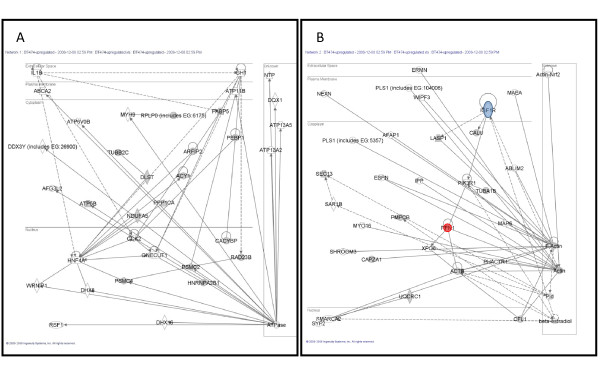
**Differentially expressed proteins for BT474 cell line were analyzed using a manually curated structured network tool (Ingenuity Pathway Analysis)**. A) Network ID 1 (*p*-value < 10^-37^) with function associated with cancer, B) Network ID 2 (*p*-value < 10^-22^) with function associated with cell morphology. White nodes are proteins identified by IPA, grey nodes are proteins identified by IPA and 2-DE, blue node is identified by IPA and validated by immunoblotting, and red node is identified by IPA and 2-DE, and confirmed by immunoblotting.

**Figure 5:**
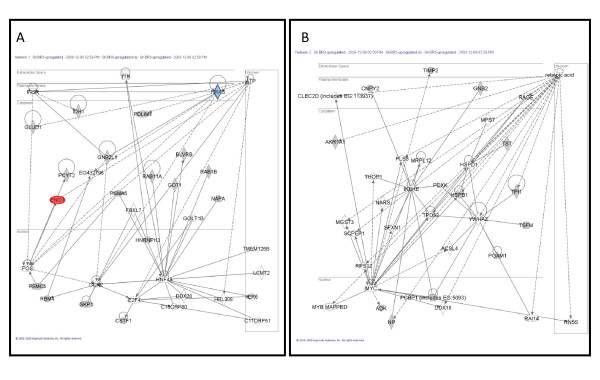
**Differentially expressed proteins for SKBR3 cell line were analyzed using a manually curated structured network tool (Ingenuity Pathway Analysis)**. A) Network ID 1 (*p*-value < 10^-42^) with function associated with cellular development, B) Network ID 2 (*p*-value < 10^-27^) with function associated with cell cycle. White nodes are proteins identified by IPA, grey nodes are proteins identified by IPA and 2-DE, blue node is identified by IPA and validated by immunoblotting, and red node is identified by IPA and 2-DE, and confirmed by immunoblotting.

These proteins were uploaded and mapped to corresponding "gene objects" in the Ingenuity Pathways Knowledge Base (IPKB). The prior information used in this analysis is a master gene interaction network curated from scientific literature. IPA then generates biological interaction networks between mapped focus genes (data set) and all other gene objects stored in the knowledge base. The IPA networks are reported in the form of a graph. The nodes of the graphs are associated with a particular gene while the edges refer to relationships identified from the literature in the IPKB. Ingenuity Pathway Analysis computes a score derived from a *p*-value, adjusted for multiple hypothesis testing, that indicates the likelihood of the focus genes in a network being observed together due to random chance. A score of 6 or greater (i.e., *p*-value < 10^-6^) indicates that there is a one in million chance of the observed subset of focus genes being observed together in a network due to random chance alone. Four networks were generated for BT474 using our data of which the top two networks shown in Figure 4 had functions associated with cancer (Figure 4A) and cell morphology (Figure 4B). Similarly, five networks were generated for SKBR3 of which the top two networks shown in Figure 5 had functions associated with cellular development (Figure 5A) and cell cycle (Figure 5B). Additional file 3, Table III shows the networks associated with BT474 and SKBR3 cell lines with the list of all proteins associated with focus genes of a network. Proteins contained within our data are highlighted in bold. The top two networks generated for BT474 exhibited a score of 37 containing 15 focus genes and a score of 22 containing 10 focus genes, respectively. Similarly, the top two networks generated for SKBR3 exhibited a score of 42 containing 17 focus genes and a score of 27 containing 12 focus genes, respectively. At the core of the network, lie one or more major hubs where multiple connections from other nodes in the network converge on or diverge into. For example, in the BT474 network there is a direct interaction between IGF-1R and PI3KR1. The hub node in SKBR3 is the HRas which has direct interactions with insulin receptor and guanine nucleotide binding protein. On the other hand, some nodes in the network figures are "hanging" (e.g. TMEM126B, LCMT2, IER5, etc. in Figure 5A) and do not form a network loop by connecting to no more than one node. Due to their association with one of the central nodes in the network (e.g. HNF4A in Figure 5A) they get "swept" up in network analysis. In Figures 4 and 5, the locations of the nodes within the IPKB generated networks are highlighted in different colors that represent how the protein was identified and confirmed.

### Validation of results by Western Blotting Analysis

**Figure 6:**
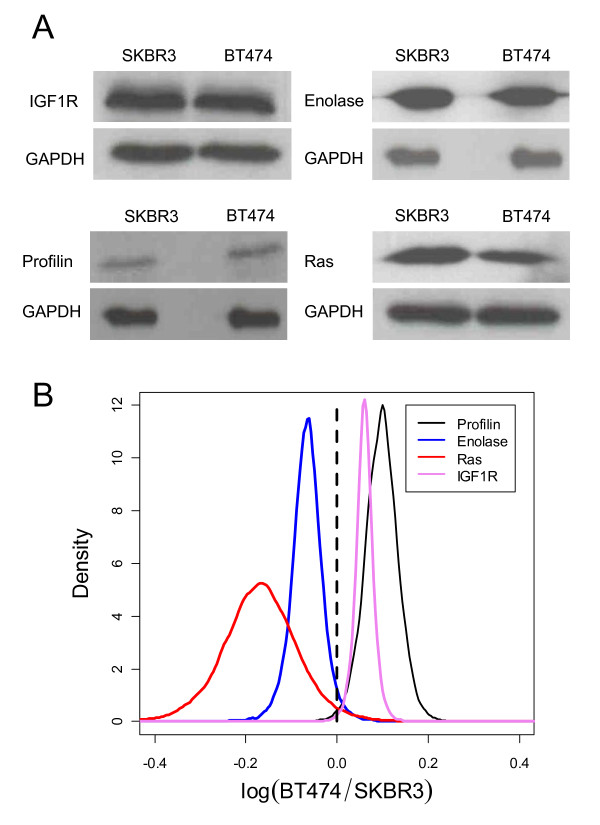
**Validating the BT-474 and SKBR-3 protein interaction networks**. A) Representative western blots for IG1R, alpha-enolase, profilin and Ras with GAPDH as loading control. B) Bayesian-MCMC estimate for the significance of differential expression within the two cell lines.

Immunoblotting was used to validate the proteomic profiling and IPA network analysis as shown in Figure 6. Two differentially expressed proteins identified by 2-DE (Profilin-1 and Alpha-enolase) and two membrane proteins in the protein network identified by IPA (IGF1R and Ras) were selected for validation. The rationale for selection of these proteins is as follows. Profilin-1 and alpha-enolase were the most differentially expressed proteins according to the proteomic data. IGF1R and Ras were selected because these proteins are the major hubs in their respective networks and to offset the bias that proteomics has against membrane proteins that limits their detectability. IGF1R has been found to be both significantly overexpressed [31-33] and highly activated [34] in cancer cells with respect to its status in normal breast tissue. Similarly, Ras is abnormally activated in breast tumors overexpressing Her2 [35]. Moreover, IGF1R and Ras are integral membrane proteins that are typically difficult to isolate and observe using 2DE [36] and occupied central positions in the protein interaction network. GAPDH was used to normalize the relative expression levels of the proteins. Western blotting analysis confirmed the general trend of expression was similar to 2-DE. Profilin-1 was up-regulated 15-fold in BT474 in cell culture as revealed by 2-DE and image analysis and up-regulated 1.4-fold (*p *< 0.01) as revealed by densitometric analysis of the western blots. Alpha-enolase was up-regulated 5-fold in SKBR3 in cell culture while maximum expectation value obtained from densitometric analysis of the western blot results suggests a 1.3-fold increase (*p *< 0.03). The western blot analysis suggests that Ras was overexpressed about 1.26-fold in SKBR3 (*p *< 0.03) and IGF1R was up-regulated 1.07-fold in BT474 (*p *< 0.001).

Findings from the western blot were in accordance with the results from proteomic analysis. IGF1R and profilin were up-regulated in BT474 and Ras and alpha-enolase were up-regulated in SKBR3. The change in density-fold from 2DE to western blots can be attributed to the semi-quantitative nature of immunoblotting as well as the differences in the dynamic ranges of photographic quantification of chemiluminiscent and fluorescent methods of detection for immunoblotting and 2-DE respectively.

## Discussion and Conclusion

It is the specific biological question that shapes the design of experiments [20]. For example, a common approach is to detect changes in expression in each spot, considered individually, that is consistently above a threshold determined by the system's experimental noise. Implicitly this work reflected a desire to strike an optimal balance between the amount of data required and the ability to infer, with predictive potential, differentially activated pathways in two cancer models. Duncan and Hunsucker have used an engineering term - fitness-for-purpose - to characterize experimental design in proteomics where different constraints; such as limits on biological samples, effective use of resources, and how the information will be used; shape the experimental design [37]. To strike a meaningful balance between these constraints, we hypothesized that a single gel replicate was sufficient to infer, with predictive potential, cell signaling pathways and protein networks that are differentially regulated between two breast cancer models. We demonstrated the predictive potential by validating the inferred differentially regulated cell signaling pathways using previously reported gene expression data [38] and by validating the inferred protein networks by western blot. Using previously reported gene expression data, we have attempted to minimize potential bias in our results introduced by our protocols (i.e., subtle differences in tissue culture or proteomics workflow). In cases where there is less prior information, such as a proteomic analysis of primary tissue, independent analysis of other gel replicates could help establish the confidence in the inferred differentially regulated cell signaling pathways. Weitzel et al. [39] recently described such an approach, where they used an additional gel replicate to confirm the upregulated pathways.

The cellular origins and predominant signaling pathways within these two cell lines are quite different. BT474 is derived from a solid invasive ductal carcinoma in the breast [40] while the SKBR3 cell line is derived from pleural effusion adenocarcinoma [41]. The aggressiveness of these cell lines are different as BT474 is ER+/PR+ with a high *in vitro *invasion capability whereas SKBR3 is ER-/PR- with a low *in vitro *invasion capability. The aggressiveness of BT474 is supported by finding that the regulation of actin based motility by Rho and actin cytoskeleton signaling pathways were enhanced. In contrast, metabolic pathways like amino acid biosynthesis and glycolysis/gluconeogenesis were more pronounced in SKBR3 cell line.

To compare our results against gene expression data for the BT474 and SKBR3 cell lines, we used the mRNA expression data from the study of a collection of breast cancer cell lines. The gene expression data was reported as a matrix of probe sets by cell lines in which value is the calculated log abundance of each probe set gene for each cell line. Gene expression values were centered by subtracting the mean value of each probe set across the cell line from each measured value. To calculate the fold-difference between the two cell lines, these log abundance gene expression values were subtracted from each other. All the up-regulated genes for BT474 (8663 genes) and SKBR3 (8237 genes) were uploaded into the IPA. As shown in Additional File 4, Table S2, the top 5 associated network functions for BT474 had an identical significance score of 27 and were as diverse as cancer, skeletal disorder and dermatological diseases. Similar analysis for SKBR3 gene expression showed the top 5 functions had an identical significance score of 25 and were as varied as embryonic development, hematological system development, and lipid metabolism. The canonical pathway analysis applied to all of the differentially expressed genes revealed that none of the canonical pathways for either cell lines were significant (Additional file 5, Figure S1). This is because the multiple-testing criteria raise the threshold for significance such that none of the embedded gene expression patterns within the dataset provide a sufficiently strong signal to surpass this increased threshold. The threshold for gene expression data was increased by setting the cut-off value to be the same as protein expression data which was 1.5-fold. The resulting analysis for BT474 dataset which consisted of 506 genes showed 4 out of top 5 associated network functions to be related to cancer with scores better than 13 as shown in Additional File 6, Table S3. The most significant pathway for this analysis was IGF-1 signaling pathway with *p*-value < 1.2 × 10^-4 ^(Figure 3B). For 304 genes which were up-regulated by a factor of 1.5-fold in SKBR3, 4 out of 5 associated network functions were related to cell death and cancer and had scores better than 11. The most significant pathway was urea cycle and metabolism of amino groups with a *p*-value < 6.45 × 10^-4 ^(Figure 3B).

The group of pathways in SKBR3 cell line associated with our protein expression data was in agreement with the group of pathways associated with gene expression data. In BT474 cell line, though the top pathways for protein expression data were associated with cell motility and the pathway for gene expression data was associated with IGF-1 signaling, they are both associated with proliferation [42] and resistance to apoptosis [43,44] in a broader sense. The number of focus molecules for the protein datasets involved in the top two networks for BT474 and SKBR3 were 25 and 29 which was 89.2% and 85.2% respectively of the total dataset; similar number for the gene datasets were 8.3% and 10.8% for BT474 and SKBR3 respectively, suggests that proteomics provides greater information per observation relative to gene expression.

In summary, experimental designs that consider each protein individually place a high burden on clinical samples. In cases where a sample is limited, a single replicate is unable to establish whether a single protein can be used as a biomarker. Our results do suggest that in a non-ideal case scenario, the overall pattern of differential protein expression can still be used, in conjunction with prior information, to infer pathways that underpin differences in cell phenotype. This information may prove helpful in tailoring therapies to the patient.

## Competing interests

The authors declare that they have no competing interests.

## Authors' contributions

YK and VS carried out the 2D-PAGE experiments. YK and VS performed the image analysis. YK was responsible for PMF, IPA analysis and immunoblotting. DK conceived of the study, participated in its design, and coordinated its execution. All authors drafted, read and approved the final manuscript.

## Pre-publication history

The pre-publication history for this paper can be accessed here:

http://www.biomedcentral.com/1471-2407/10/291/prepub

## Supplementary Material

Additional file 1 **Table II (Microsoft Powerpoint): Proteins deregulated 1.5-fold or more in both the cell lines**. Click here for file

Additional file 2 **Table S1 (Microsoft Excel): List of identified proteins labeled in Figure 1**. Click here for file

Additional file 3 **Table III (Microsoft Powerpoint): Top network associated functions generated using proteins deregulated 1.5-fold or more**. Click here for file

Additional file 4 **Table S2 (Microsoft Powerpoint): Top network associated functions generated using all up-regulated genes**. Click here for file

Additional file 5 **Figure S1 (Microsoft Powerpoint): Top 11 identified canonical pathways for BT474 (blue) and SKBR3 (red) cell lines using all deregulated genes**. The negative of the log_10_(p-value) and ratio are plotted on the primary and secondary Y-axis respectively. Click here for file

Additional file 6 **Table S3 (Microsoft Powerpoint): Top network associated functions generated using 1.5-fold up-regulated genes**. Click here for file
