# Supplementary material for: Inferring predominant pathways in cellular models of breast cancer using limited sample proteomic profiling
Source: BMC Cancer. 2010 Jun 15;10:291. doi: 10.1186/1471-2407-10-291 (PMC2896362; doi:10.1186/1471-2407-10-291)
Supplement: Additional file 1 — Table II (Microsoft Powerpoint): Proteins deregulated 1.5-fold or more in both the cell lines. [file 1471-2407-10-291-S1.PPT]

## Slide 1
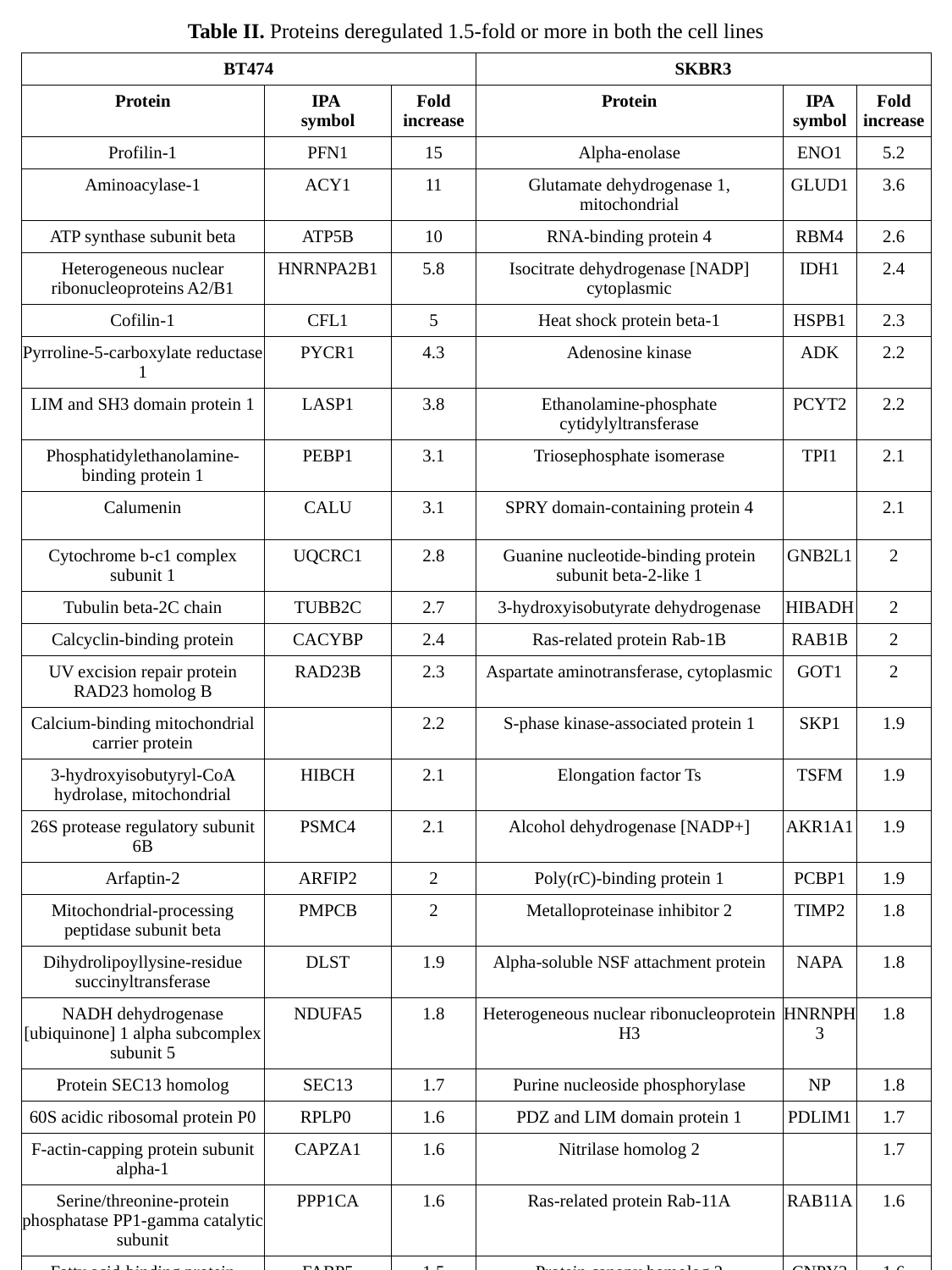

Table II. Proteins deregulated 1.5-fold or more in both the cell lines
| BT474 | | | SKBR3 | | |
| --- | --- | --- | --- | --- | --- |
| Protein | IPA symbol | Fold increase | Protein | IPA symbol | Fold increase |
| Profilin-1 | PFN1 | 15 | Alpha-enolase | ENO1 | 5.2 |
| Aminoacylase-1 | ACY1 | 11 | Glutamate dehydrogenase 1, mitochondrial | GLUD1 | 3.6 |
| ATP synthase subunit beta | ATP5B | 10 | RNA-binding protein 4 | RBM4 | 2.6 |
| Heterogeneous nuclear ribonucleoproteins A2/B1 | HNRNPA2B1 | 5.8 | Isocitrate dehydrogenase [NADP] cytoplasmic | IDH1 | 2.4 |
| Cofilin-1 | CFL1 | 5 | Heat shock protein beta-1 | HSPB1 | 2.3 |
| Pyrroline-5-carboxylate reductase 1 | PYCR1 | 4.3 | Adenosine kinase | ADK | 2.2 |
| LIM and SH3 domain protein 1 | LASP1 | 3.8 | Ethanolamine-phosphate cytidylyltransferase | PCYT2 | 2.2 |
| Phosphatidylethanolamine-binding protein 1 | PEBP1 | 3.1 | Triosephosphate isomerase | TPI1 | 2.1 |
| Calumenin | CALU | 3.1 | SPRY domain-containing protein 4 | | 2.1 |
| Cytochrome b-c1 complex subunit 1 | UQCRC1 | 2.8 | Guanine nucleotide-binding protein subunit beta-2-like 1 | GNB2L1 | 2 |
| Tubulin beta-2C chain | TUBB2C | 2.7 | 3-hydroxyisobutyrate dehydrogenase | HIBADH | 2 |
| Calcyclin-binding protein | CACYBP | 2.4 | Ras-related protein Rab-1B | RAB1B | 2 |
| UV excision repair protein RAD23 homolog B | RAD23B | 2.3 | Aspartate aminotransferase, cytoplasmic | GOT1 | 2 |
| Calcium-binding mitochondrial carrier protein | | 2.2 | S-phase kinase-associated protein 1 | SKP1 | 1.9 |
| 3-hydroxyisobutyryl-CoA hydrolase, mitochondrial | HIBCH | 2.1 | Elongation factor Ts | TSFM | 1.9 |
| 26S protease regulatory subunit 6B | PSMC4 | 2.1 | Alcohol dehydrogenase [NADP+] | AKR1A1 | 1.9 |
| Arfaptin-2 | ARFIP2 | 2 | Poly(rC)-binding protein 1 | PCBP1 | 1.9 |
| Mitochondrial-processing peptidase subunit beta | PMPCB | 2 | Metalloproteinase inhibitor 2 | TIMP2 | 1.8 |
| Dihydrolipoyllysine-residue succinyltransferase | DLST | 1.9 | Alpha-soluble NSF attachment protein | NAPA | 1.8 |
| NADH dehydrogenase [ubiquinone] 1 alpha subcomplex subunit 5 | NDUFA5 | 1.8 | Heterogeneous nuclear ribonucleoprotein H3 | HNRNPH3 | 1.8 |
| Protein SEC13 homolog | SEC13 | 1.7 | Purine nucleoside phosphorylase | NP | 1.8 |
| 60S acidic ribosomal protein P0 | RPLP0 | 1.6 | PDZ and LIM domain protein 1 | PDLIM1 | 1.7 |
| F-actin-capping protein subunit alpha-1 | CAPZA1 | 1.6 | Nitrilase homolog 2 | | 1.7 |
| Serine/threonine-protein phosphatase PP1-gamma catalytic subunit | PPP1CA | 1.6 | Ras-related protein Rab-11A | RAB11A | 1.6 |
| Fatty acid-binding protein | FABP5 | 1.5 | Protein canopy homolog 2 | CNPY2 | 1.6 |
| Actin | ACTB | 1.5 | Proteasome subunit alpha type-5 | PSMA5 | 1.6 |
| Tubulin alpha-1B chain | TUBA1B | 1.5 | Flavin reductase | BLVRB | 1.6 |
| 26S protease regulatory subunit 7 | PSMC2 | 1.5 | Cleavage stimulation factor 50 kDa subunit | CSTF1 | 1.5 |
| | | | Inorganic pyrophosphatase 2 | PPA2 | 1.5 |
| | | | Phosphoglycerate mutase 1 | PGAM1 | 1.5 |
| | | | Ribose-phosphate pyrophosphokinase 2 | PRPS2 | 1.5 |
| | | | Thiosulfate sulfurtransferase | TST | 1.5 |
| | | | 26S protease regulatory subunit 8 | PSMC5 | 1.5 |
| | | | Guanine nucleotide-binding protein G(I)/G(S)/G(T) subunit beta-2 | GNB2 | 1.5 |
