# Supplementary material for: Inferring predominant pathways in cellular models of breast cancer using limited sample proteomic profiling
Source: BMC Cancer. 2010 Jun 15;10:291. doi: 10.1186/1471-2407-10-291 (PMC2896362; doi:10.1186/1471-2407-10-291)
Supplement: Additional file 3 — Table III (Microsoft Powerpoint): Top network associated functions generated using proteins deregulated 1.5-fold or more. [file 1471-2407-10-291-S3.PPT]

## Slide 1
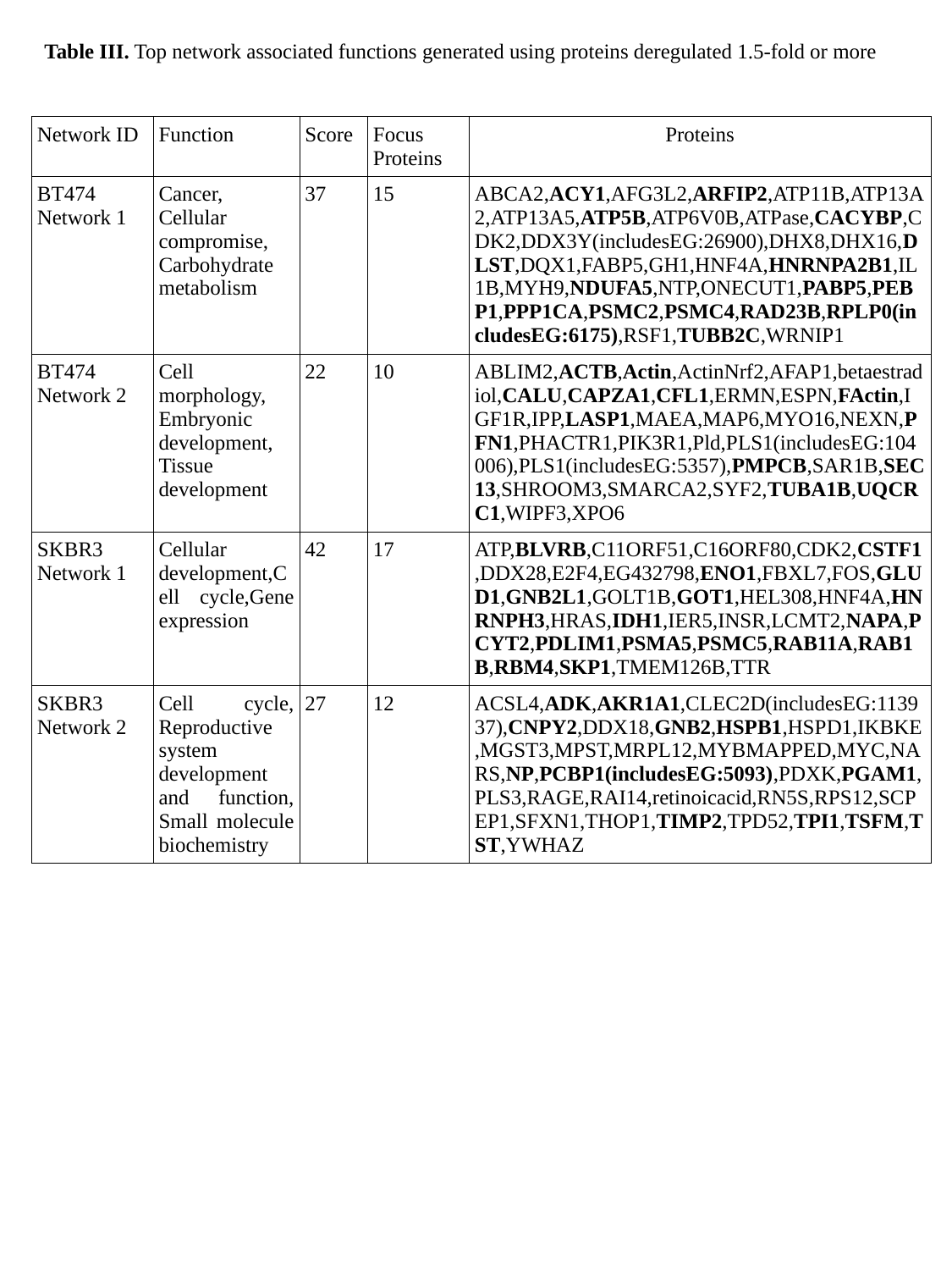

Table III. Top network associated functions generated using proteins deregulated 1.5-fold or more
| Network ID | Function | Score | Focus Proteins | Proteins |
| --- | --- | --- | --- | --- |
| BT474 Network 1 | Cancer, Cellular compromise, Carbohydrate metabolism | 37 | 15 | ABCA2,ACY1,AFG3L2,ARFIP2,ATP11B,ATP13A2,ATP13A5,ATP5B,ATP6V0B,ATPase,CACYBP,CDK2,DDX3Y(includesEG:26900),DHX8,DHX16,DLST,DQX1,FABP5,GH1,HNF4A,HNRNPA2B1,IL1B,MYH9,NDUFA5,NTP,ONECUT1,PABP5,PEBP1,PPP1CA,PSMC2,PSMC4,RAD23B,RPLP0(includesEG:6175),RSF1,TUBB2C,WRNIP1 |
| BT474 Network 2 | Cell morphology, Embryonic development, Tissue development | 22 | 10 | ABLIM2,ACTB,Actin,ActinNrf2,AFAP1,betaestradiol,CALU,CAPZA1,CFL1,ERMN,ESPN,FActin,IGF1R,IPP,LASP1,MAEA,MAP6,MYO16,NEXN,PFN1,PHACTR1,PIK3R1,Pld,PLS1(includesEG:104006),PLS1(includesEG:5357),PMPCB,SAR1B,SEC13,SHROOM3,SMARCA2,SYF2,TUBA1B,UQCRC1,WIPF3,XPO6 |
| SKBR3 Network 1 | Cellular development,Cell cycle,Gene expression | 42 | 17 | ATP,BLVRB,C11ORF51,C16ORF80,CDK2,CSTF1,DDX28,E2F4,EG432798,ENO1,FBXL7,FOS,GLUD1,GNB2L1,GOLT1B,GOT1,HEL308,HNF4A,HNRNPH3,HRAS,IDH1,IER5,INSR,LCMT2,NAPA,PCYT2,PDLIM1,PSMA5,PSMC5,RAB11A,RAB1B,RBM4,SKP1,TMEM126B,TTR |
| SKBR3 Network 2 | Cell cycle, Reproductive system development and function, Small molecule biochemistry | 27 | 12 | ACSL4,ADK,AKR1A1,CLEC2D(includesEG:113937),CNPY2,DDX18,GNB2,HSPB1,HSPD1,IKBKE,MGST3,MPST,MRPL12,MYBMAPPED,MYC,NARS,NP,PCBP1(includesEG:5093),PDXK,PGAM1,PLS3,RAGE,RAI14,retinoicacid,RN5S,RPS12,SCPEP1,SFXN1,THOP1,TIMP2,TPD52,TPI1,TSFM,TST,YWHAZ |
