# Supplementary material for: Inferring predominant pathways in cellular models of breast cancer using limited sample proteomic profiling
Source: BMC Cancer. 2010 Jun 15;10:291. doi: 10.1186/1471-2407-10-291 (PMC2896362; doi:10.1186/1471-2407-10-291)
Supplement: Additional file 4 — Table S2 (Microsoft Powerpoint): Top network associated functions generated using all up-regulated genes. [file 1471-2407-10-291-S4.PPT]

## Slide 1
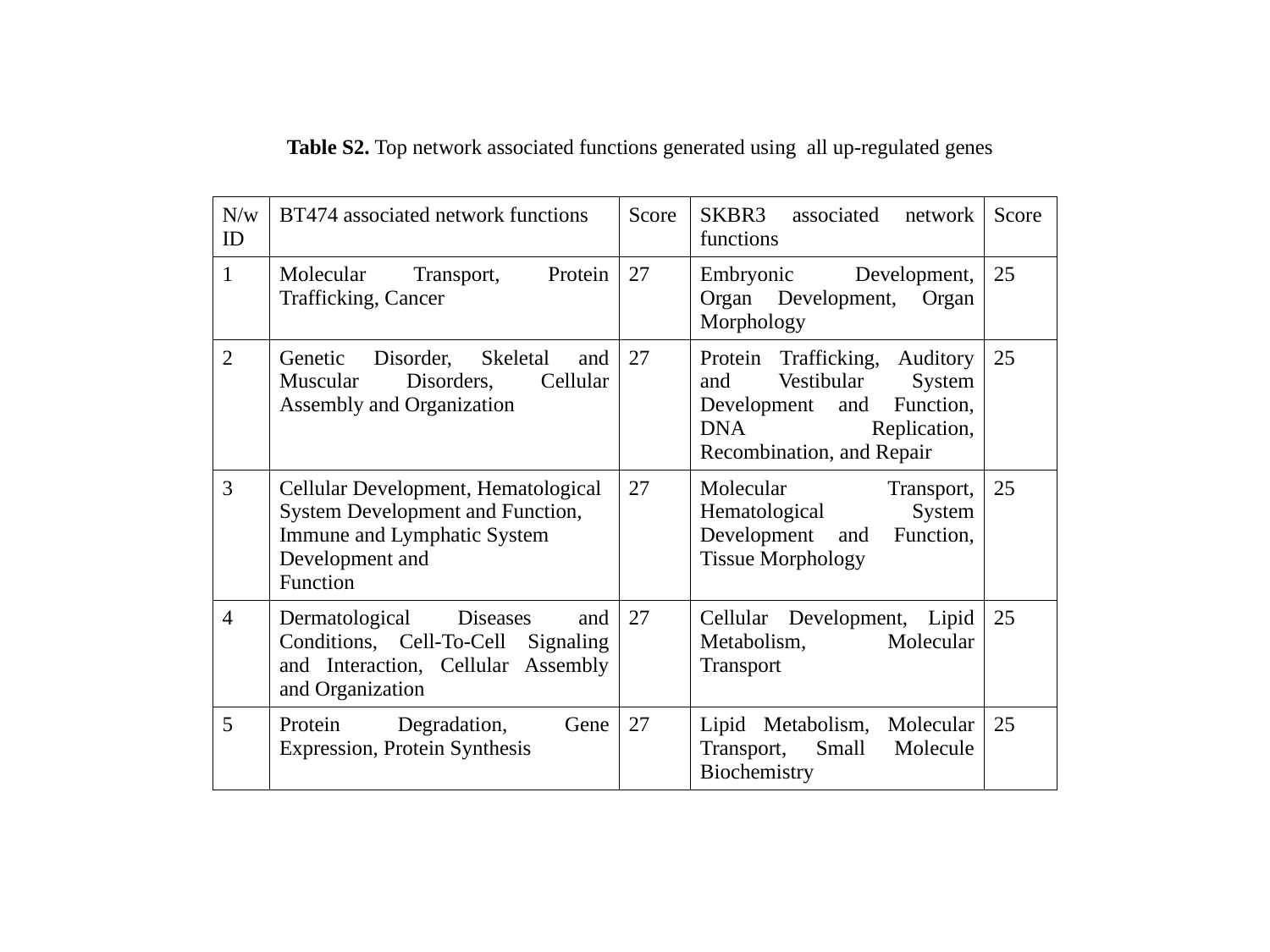

Table S2. Top network associated functions generated using all up-regulated genes
| N/w ID | BT474 associated network functions | Score | SKBR3 associated network functions | Score |
| --- | --- | --- | --- | --- |
| 1 | Molecular Transport, Protein Trafficking, Cancer | 27 | Embryonic Development, Organ Development, Organ Morphology | 25 |
| 2 | Genetic Disorder, Skeletal and Muscular Disorders, Cellular Assembly and Organization | 27 | Protein Trafficking, Auditory and Vestibular System Development and Function, DNA Replication, Recombination, and Repair | 25 |
| 3 | Cellular Development, Hematological System Development and Function, Immune and Lymphatic System Development and Function | 27 | Molecular Transport, Hematological System Development and Function, Tissue Morphology | 25 |
| 4 | Dermatological Diseases and Conditions, Cell-To-Cell Signaling and Interaction, Cellular Assembly and Organization | 27 | Cellular Development, Lipid Metabolism, Molecular Transport | 25 |
| 5 | Protein Degradation, Gene Expression, Protein Synthesis | 27 | Lipid Metabolism, Molecular Transport, Small Molecule Biochemistry | 25 |
