# Supplementary figures and images for: Inferring predominant pathways in cellular models of breast cancer using limited sample proteomic profiling
Source: BMC Cancer. 2010 Jun 15;10:291. doi: 10.1186/1471-2407-10-291 (PMC2896362; doi:10.1186/1471-2407-10-291)

## Slide 1
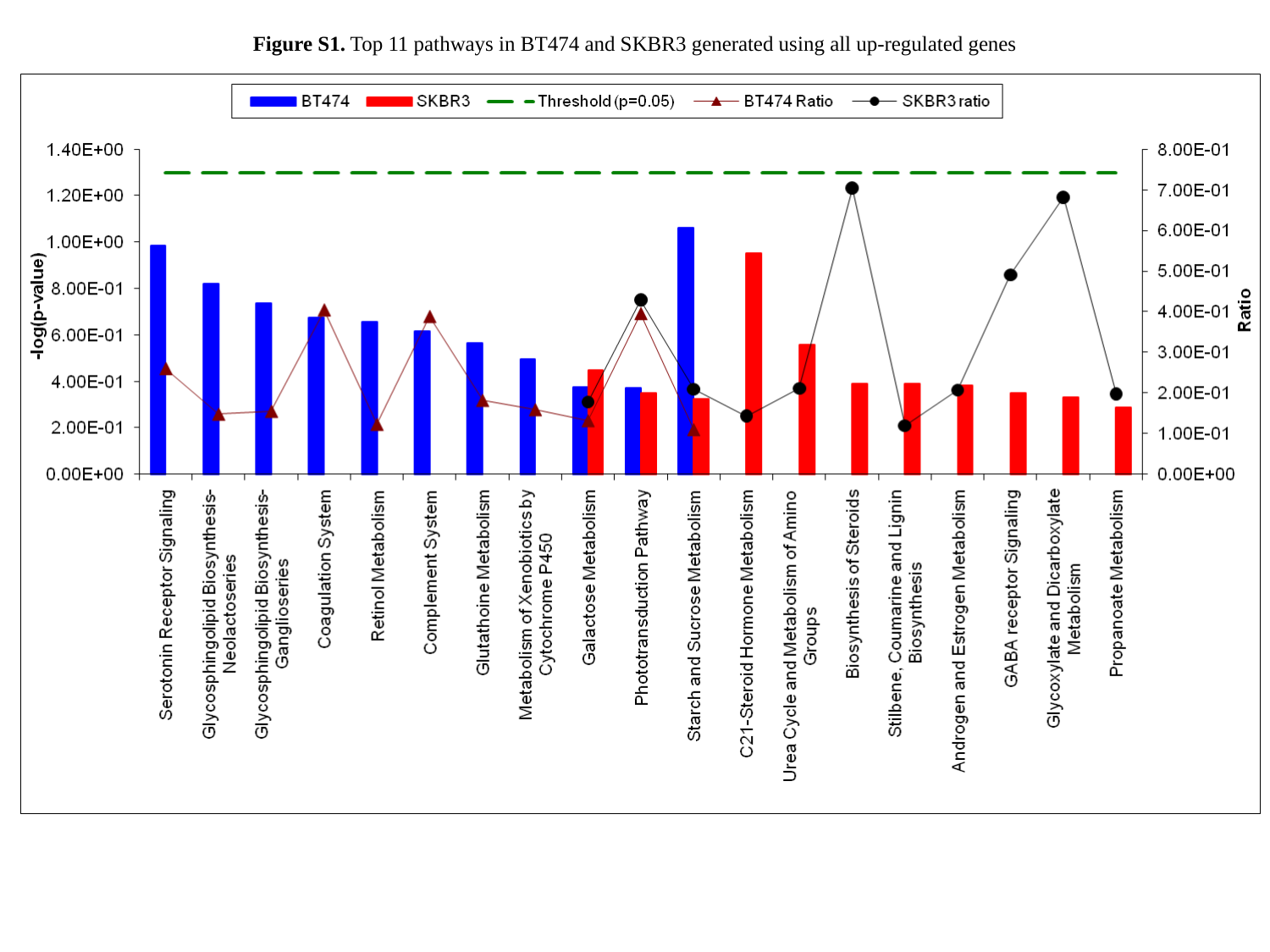

Figure S1. Top 11 pathways in BT474 and SKBR3 generated using all up-regulated genes

Supplement: Additional file 5 — Figure S1 (Microsoft Powerpoint): Top 11 identified canonical pathways for BT474 (blue) and SKBR3 (red) cell lines using all deregulated genes. The negative of the log10(p-value) and ratio are plotted on the primary and secondary Y-axis respectively. [file 1471-2407-10-291-S5.PPT]
