# Supplementary material for: Inferring predominant pathways in cellular models of breast cancer using limited sample proteomic profiling
Source: BMC Cancer. 2010 Jun 15;10:291. doi: 10.1186/1471-2407-10-291 (PMC2896362; doi:10.1186/1471-2407-10-291)
Supplement: Additional file 6 — Table S3 (Microsoft Powerpoint): Top network associated functions generated using 1.5-fold up-regulated genes. [file 1471-2407-10-291-S6.PPT]

## Slide 1
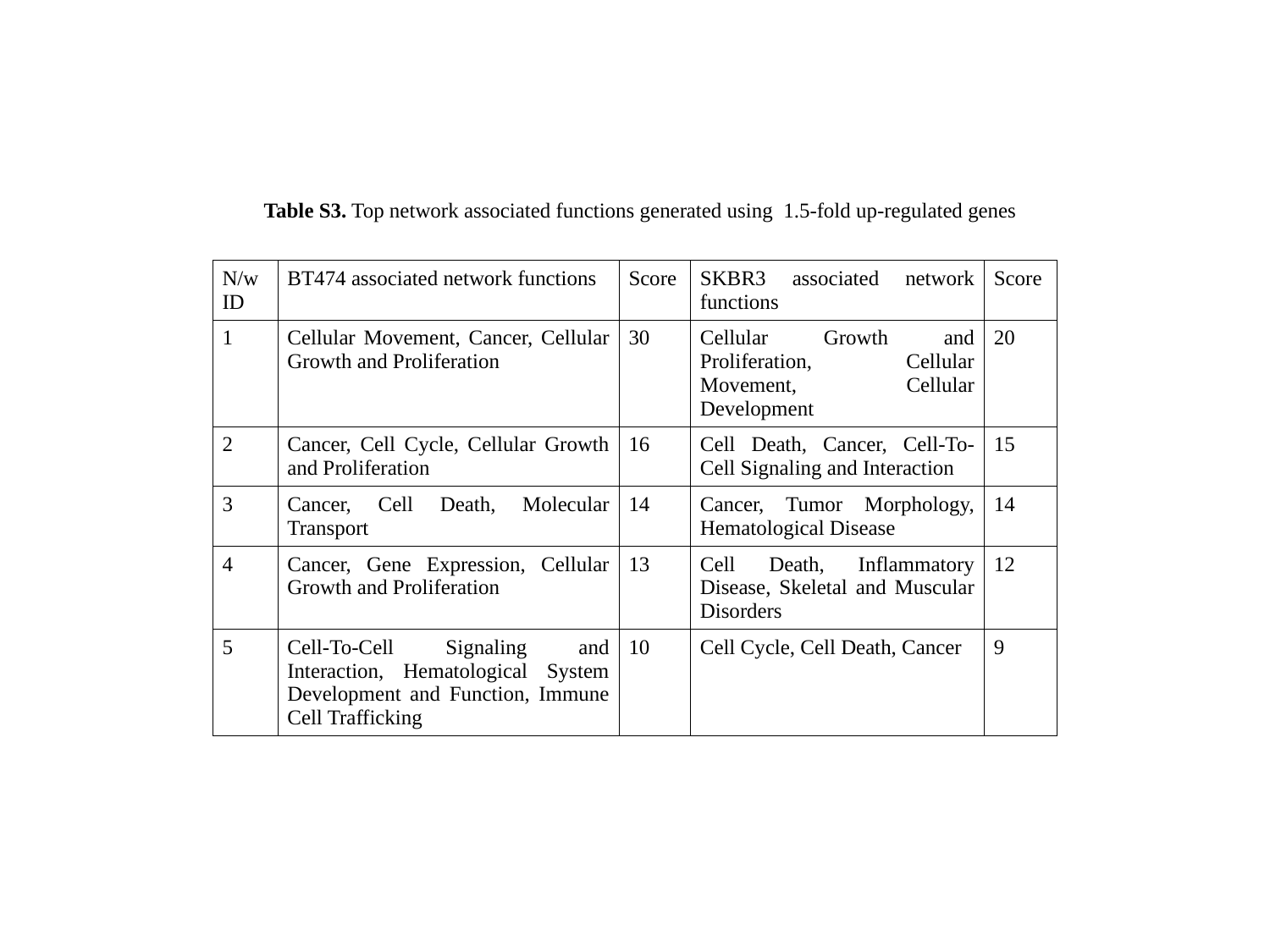

Table S3. Top network associated functions generated using 1.5-fold up-regulated genes
| N/w ID | BT474 associated network functions | Score | SKBR3 associated network functions | Score |
| --- | --- | --- | --- | --- |
| 1 | Cellular Movement, Cancer, Cellular Growth and Proliferation | 30 | Cellular Growth and Proliferation, Cellular Movement, Cellular Development | 20 |
| 2 | Cancer, Cell Cycle, Cellular Growth and Proliferation | 16 | Cell Death, Cancer, Cell-To-Cell Signaling and Interaction | 15 |
| 3 | Cancer, Cell Death, Molecular Transport | 14 | Cancer, Tumor Morphology, Hematological Disease | 14 |
| 4 | Cancer, Gene Expression, Cellular Growth and Proliferation | 13 | Cell Death, Inflammatory Disease, Skeletal and Muscular Disorders | 12 |
| 5 | Cell-To-Cell Signaling and Interaction, Hematological System Development and Function, Immune Cell Trafficking | 10 | Cell Cycle, Cell Death, Cancer | 9 |
